# The State of Person‐Centered Measurement for Family Planning Need and Use: A Scoping Review

**DOI:** 10.1111/sifp.70019

**Published:** 2025-06-11

**Authors:** Ilene S. Speizer, Elizabeth A. Sully, Youmna M. Hashem, Maame Araba A. Oduro

## Abstract

Standard measures used to assess family planning (FP) program success, including unmet need and demand satisfied, fail to capture women's, men's, couples’, and adolescents’ own expressed preferences, needs, wants, and desires. Identification is needed of person‐centered fertility and FP measures that assess what individuals want, desire, need, and prefer without a researcher‐ascribed value of what is the right outcome. This scoping review examines how the concept of person‐centeredness has been employed as part of population‐based FP measurement. For this review, we defined measures as person‐centered if they assess directly a person's preferences, wants, and desires while at the same time assessing if the person can or wants to meet those preferences, wants, and desires. The review finds several studies that use or adapt standard measures of intention to use contraception and unmet need; however, a smaller number develop novel approaches that capture method preferences and satisfaction with methods to better capture individuals’ needs, wants, and desires as well as their ability to attain these needs, wants, and desires. Results are used to make recommendations going forward for the design and testing of improved person‐centered FP measurement at the global, national, and programmatic levels.

## BACKGROUND

The Programme of Action from the 1994 International Conference on Population and Development emphasized the importance of reconsidering programming with an emphasis on meeting the fertility desires of women, men, and couples rather than focusing more narrowly on demographic targets, such as population size or fertility rates (UNFPA 2004). This revised focus aligned well with the measure of unmet need that captures the percent of women who are sexually active, have a desire to delay or avoid childbearing, and are not using contraception. The family planning (FP) field embraced unmet need and its associated indicators of demand satisfied and total demand for contraception as key demand‐side indicators included in global calls to action including the Millenium Development Goals, the subsequent Sustainable Development Goals, and the United Nations Fund for Population Assistance (UNFPA) transformative results (Speizer, Bremner, and Farid 2022). Unmet need and its related indicators were considered at the time to reflect voluntarism and informed choice because they did not focus on setting contraceptive prevalence or fertility level targets.

There is now increasing recognition that while unmet need and demand satisfied are widely used to measure program “success,” these indicators fail to capture women's, men's, couples’, and adolescents’ own expressed preferences, needs, wants, and desires. Critiques of these measures call for clarified language and definitions of existing measures as well as the need for novel measures that address equity and individual‐level preferences and behaviors (Speizer, Bremner, and Farid 2022; Holt et al. 2023; Fabic 2022; Dehlendorf et al. 2018).

To address the problems with current FP measures, there is a need to directly assess people's preferences rather than inferring them from the misalignment between contraceptive behaviors and fertility desires. The growing recognition of the importance of focusing on individuals’ contraceptive wants, needs, and desires corresponds to the current emphasis on improving the quality of health care, including FP services, through person‐centered, client‐centered, or woman‐centered strategies (Sudhinaraset et al. 2018; Afulani, Nakphong, and Sudhinaraset 2023; Holt et al. 2023; Karp et al. 2023; WHO 2007). At the same time, there is increasing attention to identifying and assessing novel person‐centered fertility and FP measures.^1^ The intention behind person‐centered measurement is clear—assessing what individuals want, desire, need, and prefer without a researcher‐ascribed value of what is the right outcome; however, there is not a consensus on how this should be operationalized or necessarily even a shared definition of what counts as person‐centered measurement.

For this review, we identify measures as “person‐centered” if they ask people directly about their preferences, wants, and desires while at the same time assessing if the person is able to or wants to meet those preferences, wants, and desires. This is consistent with a new definition of person‐centered measurement, proposed by Rothschild and colleagues (2025), though we recognize that person‐centered measurement is an emerging and growing area of new research, and there has not been a commonly agreed upon definition to date. With this definition, simply asking an individual about their self‐reported aspirations, goals, or intentions is not person‐centered as it is crucial to assess self‐identified needs and desires while at the same time determining if the individual is satisfied with the extent to which they were or were not able to meet those needs and desires (Rothschild et al. 2025). Thus, a measure such as unmet need that is externally defined based on the consistency between fertility desires and contraceptive use is not person‐centered. Likewise, intention to use contraception, while asking about an individual's preferences, is not considered person‐centered since it does not examine if a person is able to or wants to attain their reported intentions.

To date, there has been no systematic effort to comprehensively review and assess person‐centered approaches to FP need and use measurement and how these have been operationalized in population‐based research. This scoping review seeks to fill this gap by providing a synthesis of evidence on strategies and approaches used for FP measurement that seek to go beyond the standard problematic measures (e.g., contraceptive use, unmet need, demand satisfied) and begin to identify approaches for more person‐centered FP measurement.

### Objective

The present scoping review provides a comprehensive picture of how FP measurement has evolved in the last 30 years to be more thoughtful about individuals who are the intended beneficiaries. This evolution extends FP measurement beyond contraceptive use and unmet need into identifying improved strategies to better capture what individuals want, need, and prefer. We identify if the study is seeking to adapt existing measures to be “more person‐centered” and if these measures meet the criteria of person‐centeredness that we set out above, or if the study has specifically developed novel person‐centered FP measures. Findings from the review are used to make recommendations for going forward to develop new measurement frameworks and indicators to help advance how population‐based research and program evaluations capture information on crucial aspects of equity, justice, rights, perceived needs and preferences as they pertain to women's, men's, couples’, and adolescents’ FP attitudes and behaviors.

This review offers information on measurement progress to date to identify person‐centered FP need and use measures and is part of a process to get to improved global, national, local, and programmatic measurement. While the goal is to have more person‐centered FP measures in the future, there is also the recognition that measures may exist on a continuum of more person‐centered to fully person‐centered, and different measures may be more appropriate for different circumstances. Notably, important measures for program monitoring may not be person‐centered, but, as proposed by Rothschild and colleagues (2025), they should be rights‐ and justice‐based measures. However, after decades of measurement focused on researchers (or program/donor) ascribed desired outcomes or assumptions on what people want or need, this review focuses on the critical shift taking place in the FP measurement field toward more person‐centered measures.

## Methods

This scoping review followed the standard steps of a systematic review, although necessary modifications were made along the way, given that the topic did not fit cleanly into the typical structure of population, intervention, comparator, and outcome (PICO) review. For this reason, we undertook a scoping review rather than a systematic review. The following steps were taken and are described in more depth below: (1) finalize research focus; (2) undertake search of literature; (3) undertake title and abstract review; (4) undertake full text review; (5) extract relevant information; and (6) present and interpret the findings.

*Step 1: Finalize the research focus*. The research emphasis for this analysis is on identifying person‐centered concepts that have influenced the measurement of FP preferences and behaviors globally. With this in mind, we wanted to know how these concepts were operationalized and how they have been used to influence programs and policy in both low‐ and middle‐income countries and high‐income countries. We also wanted to identify gaps in person‐centered measurement. Notably, given the importance of unmet need (and demand satisfied) as key measures in the global FP field, we expected to find many studies using these indicators, but our objective was to go beyond these measures and thus we sought studies that adapted these measures or proposed alternative measures.

*Step 2: Search strategy*. This was a complex step since we were seeking studies that included “person‐centered” measures around FP attitudes and behaviors. Given the novelty of the use of person‐centered language, specifically around person‐centered health care or person‐centered reproductive health (Sudhinaraset et al. 2017), the search terms that we were focused on were not necessarily cleanly defined. With that in mind, we included search terms in four main categories: (a) subject area, (b) context, (c) key concepts, and (d) person‐centered. Below is a table that summarizes search terms that fit into these categories. The timeframe of focus was publications from 1990 through 2023. While we were prepared to review articles in English, French, and Arabic (given the structure of the team), all articles that were identified and found were in English. The search happened in three databases: PubMed, Scopus, and Global Health. All information was brought into Covidence for review and assessment.

**Table:** 

Category	Search terms
Subject area	family planning, contraception, contraceptive, fertility, abortion, pregnancy, pregnant, childbearing, postpartum, birth control
Context	indicator, indicators, measure, measurement, evaluation, evaluations, evaluate, research, researches, monitor, monitoring
Key concepts	intent*, intend, prefer*, desire*, demand*, unmet need, met need
Person‐centered	person centered, person‐centered, client centered, client‐centered, woman centered, woman‐centered, patient centered, patient‐centered, person centred, person‐centred, client centred, client‐centred, woman centred, woman‐centred, patient centred, patient‐centred, rights based, rights‐based, autonomy, agency, volition, reproductive justice, empower*

NOTE: Terms with a “*” mean that we searched iterations of the word (e.g., intent, intention; prefer, preference).

**FIGURE 1:**
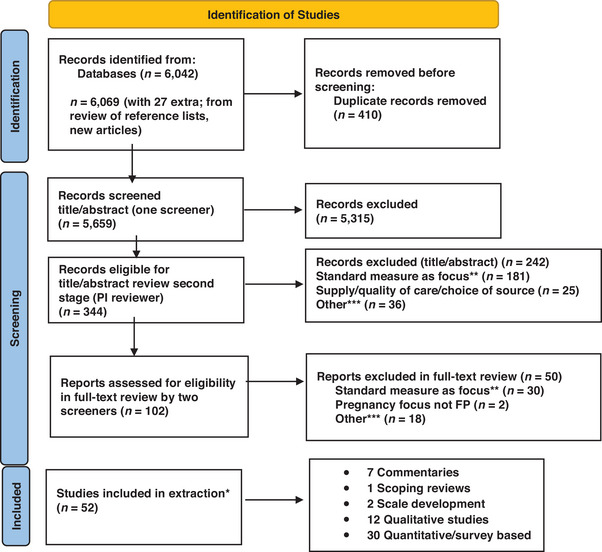
Prisma flow diagram for scoping review of person‐centered family planning measurement NOTES: *Sixteen out of 52 studies only had the principal investigator (PI) do extraction; this person did extraction on all 52 studies. **This includes studies that use standard measures of unmet need, contraceptive use, demand satisfied, intention to use, fertility desire, and qualitative studies on similar/related topics. ***This includes studies that were out of scope, including those on program interventions, intimate partner violence, particular to a special population, focused on abortion decision‐making, focused on person‐centered service delivery, etc.

It is important to note that while this search led to more than 6,000 articles, there were some identified papers that were missing that needed to be incorporated based on reference lists of some of the papers or new papers recently released in 2024. Twenty‐seven papers were added to the search from these alternative approaches. (See Figure 1 for the Prisma diagram of the studies reviewed.)

*Step 3: Title and abstract review*. For this search, two levels of title and abstract review were undertaken as shown in the Prisma diagram. The project team initially jointly reviewed a small set of articles to determine the scope of what was the focus. This included two graduate students (third and fourth authors) and the principal investigator (first author). After the joint review, each member of the review team reviewed the same five articles separately and compared their results. This helped to standardize the review process. Once the team was reviewing similarly, the first stage of title and abstract review was conducted by the two graduate students (one reviewer per article). When they had articles that they were not sure about whether to include or not, these were discussed jointly during regular team meetings. From the full set of 5,659 articles for title and abstract review, there were 5,315 excluded. The remaining 344 articles included measures related to fertility and FP, including unmet need, met need, demand satisfied, and contraceptive use. As a second title and abstract review step, the principal investigator (PI) reviewed all 344 articles and categorized them into four categories: (a) potentially person‐centered measures of interest; (b) standard measures with no modifications (e.g., unmet need, contraceptive use, met need, demand satisfied, and qualitative studies on similar topics); (c) supply or quality of care or source focused; and (d) other reason for exclusion. This last category included studies that were deemed out of scope, including program interventions, focus on intimate partner violence, focus on the choice of a specific method (e.g., emergency contraception), focus on a special population and their needs, or focus on person‐centered service delivery. This resulted in removing 242 articles, the majority of which used the standard measures of unmet need, met need, contraceptive use, etc. (see Figure 1, Prisma diagram).

*Step 4: Full text review*. At this step, there remained 102 articles for full text review. Again, the team jointly reviewed full text for a small number of articles and used these to test out our selection criteria for what type of studies remained in the pool and which ones were dropped. Once we were reviewing similarly, one graduate student reviewed each of the studies, and the PI reviewed all of the studies as the second reviewer. Upon completion of the full text review, we compared cases where we differed in our determination of whether the studies were applicable to our focus. For those that were dropped from consideration after full text review, we also coded them into similar categories as those excluded above. Out of the 102 studies reviewed, 50 were excluded with 30 of them simply focusing on standard measures, two focused on pregnancy desires, and 18 were in the other category that were deemed out of scope for this review.

*Step 5: Extraction*. Extraction was undertaken using a standardized form developed by the study team. Among the 52 articles reviewed for extraction, 36 were reviewed by two reviewers (one of the students and the PI), and the last 16 articles were extracted solely by the PI, given that the student workers had completed their time on the project. The extraction identified studies into five categories: seven commentaries, one scoping review, two focused on scale development,^2^ 12 qualitative studies, and 30 quantitative/survey‐based studies. Of note, while the 52 studies were related to the topic, a number do not make specific recommendations for person‐centered FP measurement (e.g., qualitative studies) or simply propose that this is needed (commentaries). Also, some of the quantitative or survey‐based studies propose novel person‐centered measures, while others are adaptations to existing measures to be “more person‐centered” but do not meet the classification of person‐centered as defined above. This is discussed in the Results section below.

### Limitations

Several limitations were identified throughout the search process. First, the lack of standardized language used around person‐centered FP need and use measurement when this review was underway was a challenge. With that in mind, it is likely that we missed some relevant articles on the topic. Because of this, we included some additional papers based on the reference lists of identified papers and recent papers that were published in 2024, as noted above. That said, we still expect we missed some relevant papers, including recently published papers, given the rapid growth in this new area of research. Second, given the novelty of person‐centered measurement, the review included papers that seemed to fit but at the extraction phase fit less well; these are included here but do not significantly inform the recommendations. Third, we identified several studies that examined measures related to agency, autonomy, empowerment, and FP decision‐making and contraceptive use. A number of these were excluded as they focused more broadly on reproductive autonomy rather than fertility or FP (e.g., Upadhyay et al., 2014), or they used standard decision‐making items to predict standard FP outcomes of contraceptive use or unmet need. For readers interested in a more detailed review of agency in FP and reproductive health, the recent scoping review by Wood and colleagues (2025) is a good resource.

## Results

In reviewing the papers that were included for extraction, we were able to categorize them by the domains of contraceptive use as well as by the stage of measurement development (see Table 1 and Online Appendix A). The domains of contraceptive use include (a) decision‐making/agency/autonomy; (b) contraceptive use intentions or unmet need‐related; (c) pregnancy and fertility preferences; (d) method preferences; and (e) method experience and satisfaction. In terms of the stages of measurement development, the papers fell into the following categories: general conceptual papers; specific conceptual papers; formative studies; scale development; validation of measures; and applied studies. This last category was divided into those that employed cross‐sectional data and those that employed longitudinal data and methods.

**TABLE 1:** Number of studies by the relevant domains of contraceptive use and measure development stages

Measure development stages	Domains of contraceptive use				
	Decision‐making/agency/ autonomy	Contraceptive use intentions/unmet need related	Pregnancy/fertility preference	Method preference	Method experience/satisfaction
Conceptual: General	Three commentaries				
Conceptual: Specific concept	One commentary	Three cross‐sectional data		Two Commentaries	One commentary
Formative	Five qualitative	One scoping review One qualitative	Three qualitative	Three qualitative	
Scale development	Two cross‐sectional data	One cross‐sectional data		One cross‐sectional data	
Validation			Three cross sectional		One cross‐sectional data
Applied: Cross‐sectional Applied: Longitudinal	One cross‐sectional data	Six cross‐sectional data Seven longitudinal data	Three cross‐sectional data	Three cross‐sectional data One longitudinal data	Two longitudinal data

Below, we present the results in broad categories. We first summarize the recommendations from three overarching commentaries related to the importance of novel measures that are more person‐centered or at a minimum, consider domains important to women, men, and adolescents and not just researchers. Second, we briefly summarize what we know from the 12 qualitative studies that are related to person‐centered considerations about decision‐making, agency, and autonomy, among other areas. Third, we briefly discuss two studies that present scale development related to improved contraceptive decision‐making measurement. Finally, based on the 30 studies that used survey or quantitative data as well as a scoping review on intention to use contraception, we provide a summary of findings and recommendations by the focus of the study. In this latter section, we weave in four additional commentaries that make specific recommendations for revised or adapted measures related to contraceptive use preferences (Holt et al. 2023; Burke and Potter 2023), satisfaction with methods (Rominski and Stephenson 2019), and autonomy (Senderowicz 2020). New measures have been developed in direct conversation with the recommendations from these commentaries and together represent important domains of new measure development in the field.

### High‐Level Commentaries

Three commentaries recommend rethinking our FP measurement approaches. Two commentaries take a higher level framing and push for clarifications in terminology used (Fabic 2022; Speizer, Bremner, and Farid 2022) as well as consideration of specific needs, wants, and desires with a reproductive justice and rights framing (Speizer, Bremner, and Farid 2022). Further, a commentary by Dehlendorf and colleagues (2018) demonstrates that the research questions that are asked and the indicators that are selected can impact whether women's autonomy is being prioritized. The authors promote the need for thoughtful framing of research questions and selection of indicators that reflect person‐centered or patient‐centered perspectives; they acknowledge that this may require additional formative research that helps to determine how best to measure what matters to individuals. These commentaries have been key for pushing the field, both in the United States and globally, to begin questioning what we are measuring and why we continue to measure using the standard, flawed measures that fail to consider what individuals want, need, desire, and prefer. These commentaries have served as both a call‐to‐action for the field and represent the growing momentum taking place in the last half a decade toward person‐centered measurement.

### Qualitative/Formative

The 12 qualitative studies included in the extraction phase mostly focused on understanding reproductive autonomy and decision‐making among young women (*n* = 8). Two additional studies examined issues of agency and autonomy and method choice among women and men (or couples), and two others were among postpartum women (see Table 2).

**TABLE 2:** Summary of qualitative studies included in the review

First author year	Location	Research objective or hypothesis	Study sample and approach	Person‐centered measures/concepts	Main results/lessons learned	Relevance to measurement
Studies focused on young women						
Harrington 2021	Kenya	Examines how Kenyan adolescent girls and young women (ages 15–19) perceive FP needs and make FP decisions within their social contexts	Forty in‐depth interviews (IDI) and 46 participants in 6 focus group discussions (FGD) (ages 15–19)	Interplay between social environment and perceived self‐efficacy on decision‐making	Contraceptive use is viewed as against social norms but young people choosing to use it in spite of social pressures; young people risk stigma through FP use or pregnancy experience	Focus on young people's decision‐making agency.
Paul 2017	India	Explores how young women practice fertility control in a context where they have low autonomy. Focuses on reproductive intentions, desires, and choices available to women in relation to social expectations and accessible health services	Twenty‐four IDI with young women ages 18–24 (focus on recently married)	Autonomy, freedom, and restrictions within and before marriage	Changes in notions of agency across generations. Recent social changes have created unique opportunities for young women's influence in reproductive decision‐making. New “agentic” opportunities that enable women's individual reproductive decision‐making. Two types of agency are identified: collective and individual	Important to consider reproductive agency
Willan 2020	South Africa	Explore which young women exercise reproductive decision‐making and avoid early motherhood, and why, and what enables them to do so, and to examine the experience of young women who conceive early	IDI with 15 young women (ages 18–30) interviewed at three time points	Reproductive agency and decision‐making	Normative pressures affect early conception—no plan to conceive and do not make reproductive decisions. Young women with children focus on spacing and timing of future children and delaying future pregnancies. Inconsistent and ineffective contraceptive use because of misinformation and myths, and fear of being mistreated by healthcare providers	Social norms theory is important at younger ages, while agency theory is important later
Hayer 2022	South Africa	Examines young women's access to and use of contraception to understand how their contraceptive intentions are formed	IDI with 14 women ages 18–28	Contraceptive preferences and intentions	Agency enacted within relationships (partner/family) in which gendered expectations shape method preference and use. For young women, uncertainty and concern coexisted with ideas of agency and prevention	Consider relationships and personal constraints to better understand if young women are fulfilling their personal reproductive goals
Burgess 2022	Côte d'Ivoire	Examine the reproductive health needs of women who aspire to start childbearing in uncertain circumstances	IDI, FGD, and daily diary with 20 young (18‐30), unmarried women; two‐week follow‐up	Fertility desires and contraceptive use	Improvised use of FP methods related to ambiguous fertility desires and concerns about future fertility	Consider the multiple pressures that young, unmarried women experience
Downey 2017	United States	Examine contraceptive decision‐making processes & perspectives on IUDs among young women at high risk of unintended pregnancy	IDI with 38 young (ages 18–24) Black and Latina women	Influences on contraceptive decision‐making, such as preferences, personal values, and relationship status	Pregnancies and childbirth were pivot points; women often initiate a change in their method or return to a method following a pregnancy and/or childbirth	Consider preferences, personal values, and relationship status
Berglas 2021	United States	Examine young women's decisions to use lower efficacy contraceptive methods.	IDI with 22 young (< 30) African American and Latina women seeking emergency contraception (EC)	Decision‐making and motivations	Young women's decisions about contraceptive use are informed not only by their desire to prevent pregnancy but also by other affirmative values	Consider young women's reproductive autonomy and choice of using less effective methods
Gomez 2021	United States	Explore how structural factors and systemic racism influence young people's ability to make decisions related to their reproductive preferences	IDI with 50 young (18 = ‐24) women and their male partners	Decision‐making concerning reproductive preferences	Structural inequities influence the path from envisioning to actualizing reproductive desires	Consider structural inequity for counseling young people since it affects contraceptive and reproductive preferences
**Studies focused on women and men**						
Grillo 2018	Uganda	To understand how couples perceive a recent unintended pregnancy in the context of HIV infection, where there are high levels of gender inequality	IDI with 12 male and female partners, where one or both reported an unintended pregnancy	Agency and how it affects decision‐making	Agency and the complex spectrum from control to lack of control found by female and male partners	Relevance of dyadic data and analysis to understand agency
Osei 2014	Ghana	Examine factors at individual, interpersonal, and social levels to understand the contexts in which men and women make contraceptive decisions through their reproductive lives	IDI with 26 men and 54 women	Decision‐making around FP use and choice of traditional methods	Relationship characteristics affect FP decision‐making—the method used tended to depend on the type and stage of the relationship, and by changing social norms regarding premarital sex and women's autonomy within relationships. The traditional method use was a choice	Consider relationship characteristics and traditional methods as a preferred option
**Studies focused on postpartum women**						
Spagnoletti 2018	Indonesia	Focuses on the normative social expectations and relational factors including interactions with the health system that influence decisions of postpartum women in relation to FP	Thirty‐one IDI with 20 women who delivered three weeks—18 months prior	Reproductive agency	Reproductive agency is limited by information provided by family and friends; public sector providers’ coercion and misinformation also affect reproductive agency	Consider reproductive agency to better understand decision‐making among postpartum women
Spagnoletti 2019	Indonesia	Examines why middle‐class women in urban Yogyakarta is choosing nonbiomedical and nonhormonal contraceptives, and determining how women explain their intent to use these methods during the postpartum period	Thirty‐one IDI with 20 women who delivered three weeks—18 months prior	Reproductive agency and choice of method	Contraceptive choices indicative of reproductive modernity and agency and go against the state‐driven population control focus. High degree of reproductive agency in terms of their negotiations with husbands.	Consider reproductive agency to better understand decision‐making among postpartum women

NOTE: FP, family planning; FGD, focus group discussion; IDI, in‐depth interview; IUD, intrauterine device.

The papers that examined decision‐making among adolescents and youth identified the role of social norms in affecting decisions and behaviors, particularly for younger and unmarried women (Harrington et al. 2021; Willan et al. 2020; Hayer et al. 2022; Burgess et al. 2022). These same young people are also influenced by misinformation and myths as well as provider behaviors that may limit their ability to use contraception when or if they choose to (Willan et al. 2020; Harrington et al. 2021; Hayer et al. 2022; Downey et al. 2017). That said, some adolescents refer to the importance of self‐protection and demonstrate agency to avoid unintended childbearing; this was more common among the older adolescents and youth who were less influenced by social norms (Willan et al. 2020; Harrington et al. 2021; Paul et al. 2017; Hayer et al. 2022; Downey et al. 2017). There was also a tension that was discussed between gendered expectations and norms and actual behaviors for self‐preservation (i.e., agency) (Hayer et al. 2022; Paul et al. 2017). The role of structural inequities was identified as affecting young people's ability to actualize their reproductive desires; this was a particular issue raised in a study from the United States (Gomez, Arteaga, and Freihart 2021). Further, some young women are explicitly choosing to use less effective methods with the idea that this better meets their sexual behaviors and needs (Berglas et al. 2021). This was also found among adult women and men (Osei et al. 2014) and among postpartum women who choose nonhormonal and nonbiomedical methods (Spagnoletti et al. 2019).

Papers reporting on studies among women and men, including couples, demonstrate the importance of agency and acknowledge that there is a spectrum of agency that varies across couples and over the stages of a relationship or life course (Grillo et al. 2018; Osei et al. 2014). Further, among postpartum women in Indonesia who were the focus of the work by Spagnoletti and colleagues (2018, 2019), the authors demonstrate gaps in reproductive agency influenced by persons who control postpartum women's access to and understanding of information, including family, friends, and health care providers.

The qualitative papers provide formative information to demonstrate the importance of understanding in more depth the role of agency and autonomy in influencing FP decision‐making. Some also point out that the choice of traditional methods or less effective methods needs to be examined as a rationale choice made by individuals (i.e., a person‐centered choice). Further, some of the papers pushed for the importance of obtaining information from both women and men (i.e., couples or not) to better understand the relational context within which decision‐making is happening. That said, none of the papers make specific recommendations for improved person‐centered FP measurement. This type of formative work is crucial for identifying the domains of person‐centered FP measurement that need to be considered going forward. These include considering agency and autonomy, examining whose opinion matters for decision‐making measurement, and reflecting on the methods that are considered relevant and important to users or future users of contraception. Notably, many of these considerations show up in the quantitative survey‐based papers identified.

### Scale Development

Two extracted papers focused on scale development for improved measurement of individuals’ FP needs. The paper by Carvajal and colleagues (2020) focused on developing ethnically responsive, content‐valid, theory‐based questions to measure the personal and health care factors that influence contraceptive decision‐making among immigrant Latina women. Raine‐Bennett and Rocca (2015) test a novel tool, the Contraceptive Intent Questionnaire, to assess the conscious and unconscious factors that relate to risk of contraceptive nonadherence (i.e., failure to use a chosen contraceptive method) among young (ages 15–24), female FP clients from the United States. Both scales are theory based. The scale by Carvajal and colleagues (2020) includes 27 items related to influencers (teachers and friends) as well as preferences (i.e., contraceptive preference), interactions with providers (communication, trust, language, sex of provider, nonjudgmental and nondiscriminatory), knowledge about methods, and confidentiality. The scale by Raine‐Bennett and Rocca (2015) includes 15 items that are reliable and valid for use by U.S. providers with young women to help identify and counsel them on contraceptive use intentions and decision‐making. While both scales seek to identify what individuals want, need, or prefer, they are both targeted to improved counseling of clients and thus most relevant for U.S. clinic settings rather than broader, population‐based measurement of FP needs and use.

### Quantitative Survey‐Based Studies

As shown in Table 1, the 30 quantitative survey‐based studies had the most emphasis on contraceptive use intentions and adaptations to the unmet need measure as the main domain of interest using cross‐sectional or longitudinal data. The next most common domain of focus was method preference, with some cross‐sectional and a couple of longitudinal studies. The remaining studies covered the domains of pregnancy and fertility preference, and method experience or satisfaction. For this analysis, we present the results of the quantitative survey‐based studies as follows. First, we discuss intention to use focused studies, including summarizing a scoping review on intention to use; we place these studies into the context of how they have influenced the field to be more responsive to individuals’ desires and preferences, but do not attain the objective of being person‐centered. Second, we talk about studies that have sought to adapt standard measures of unmet need, met need, and demand satisfied. These studies are typically seeking to address faulty assumptions of the standard measures but often fail to be fully person‐centered in their final approach. Third, we present a small number of studies that focus on approaches to measure pregnancy or fertility desires that recognize the complexity of decision‐making. Finally, we examine four studies that present novel, person‐centered FP measures (see Table 3).

**TABLE 3:** Summary of survey‐based quantitative studies included in the review

First author year	Location(s)	Research objective or hypothesis	Study design	Study population^a^	Person‐centered measures or concepts	Main results/lessons learned	Relevance to measurement
**Studies focused on the intention to use as a key component**							
Hanson 2015	United States	Examine direct and indirect measures of perceived behavioral control (PBC) related to contraceptive use; determine how PBC is related to intention to use and actual use	Cross‐sectional: Mail survey	190 nonpregnant patients ages 18–44 in the health system in the last 3 years	Direct PBC for birth control; indirect PBC (i.e., control questions and power questions) and intention to use (expect to use, want to use, and intend to use)	Indirect and direct PBC measures correlated; a significant and direct relationship of PBC and birth control behavior, even when intention is added to the model	Perceived behavioral control is an interesting measure but not specifically defined as person‐centered
Gage 2021	Democratic Republic of Congo	Determines whether perceived norms and personal agency predict postpartum FP (PPFP) intentions among first‐time mothers (FTM) ages 15–24	Cross sectional	2,431 first‐time mothers ages 15–24 pregnant with their first child	The PPFP intentions scale captured five likely components of the decision‐making process	FTMs with higher personal agency (control over posuse; self‐efficacy) or PPFP autonomy had significantly higher PPFP intentions than those who lower personal agency or PPFP autonomy	Discusses the importance of PPFP intentions among FTM; not a novel person‐centered measure
Khan 2023	Nigeria	Develop and test a new index of empowerment and determine if it is related to the intention to use contraception after marriage	Cross sectional	240 unmarried girls ages 13–19 in rural high schools	Existence of choice—power relations with guardians and gender norms	In the adjusted model, perceived career feasibility was the only factor significantly related to intentions to use contraception in the future	Consider girls empowerment as part of the measurement of intentions
Atiglo 2019	Ghana	Examine the extent of women's household decision‐making autonomy and its relation to meet the demand for contraception compared to no intention to use	Cross sectional	1,971 women ages 15–49, married or in union, and sexually active in the last 4 weeks not currently pregnant or trying to get pregnant	Decision‐making autonomy was assessed by responses to five questions about involvement in decision‐making	Moderate and high levels of autonomy were associated with over 1.5 times the likelihood of having a met demand rather than having no intention for use. Among women with contraceptive demand, household decision‐making autonomy is minimally associated with contraception uptake	Autonomy in the dimension of household decision‐making may not necessarily predict reproductive behavior; important to consider intentions to use
**Studies focused on intention to use (and unmet need status) and subsequent use (longitudinal)**							
Callahan 2014	Bangladesh	Examine the relationship between intention to use and subsequent use by unmet need status to determine who is more likely to use over time	Cohort study	3024 women ages 13–49 interviewed at baseline and three‐year follow‐up	Unmet need plus intention to use contraception	Many women moved between categories of unmet need and contraceptive use between 2006 and 2009	Focus on unmet need to assess contraceptive demand and risk of unwanted pregnancy among nonusers results in missed opportunities for meeting the needs of existing users, including those using less effective, traditional methods who may be at risk for contraceptive failure or discontinuation
Lutalo 2018	Uganda	Examine rates and factors associated with unfulfilled need for contraception among women who expressed an intention to use	Cohort study	2610 nonpregnant, mostly rural women ages 15–49 with an unmet need (up to seven‐year follow‐up)	Unfulfilled need for contraception based on baseline intentions and pregnancy experience over follow‐up	The majority (57‐ 65 percent) who intended to use at first observation had an unfulfilled need at follow‐up; however, the proportion intending to use increased	The study states that unmet need is an inaccurate measure
Borges 2018	Brazil	Examine the intention to use among postpartum women and the subsequent use six months later	Cohort study	474 pregnant women ages 15–44 enrolled and 256 completed six months postpartum follow‐up interview	Concordance between contraceptive preference and use coded as yes if a woman is using at six months’ follow‐up the method she preferred when she was pregnant	Women who achieved contraceptive preference‐use concordance had a higher proportion of planned pregnancy. Women who were not sure about what method they intended to use after childbirth more often reported no use at six months postpartum. Less than one third of postpartum women were using the method that they reported they intended to use after childbirth	Assessing pregnancy intentions should be incorporated into routine health professional practices. Pregnancy planning status is associated with postpartum contraceptive preference‐use concordance
Roy 2003	India	Examine how well‐stated contraceptive use and childbearing intentions relate to later behaviors	Cohort study	432 women of reproductive age not sterilized at the first interview, with a follow‐up interview six years later	Gap between fertility intentions and contraceptive intentions	Contraceptive intentions appear to be better predictors of behavior than are childbearing intentions. May need to examine contraceptive and fertility intentions jointly	Use contraceptives and fertility intentions jointly
Curtis 1996	Morocco	Measure the relationship between intention to use contraception and subsequent contraceptive use	Cohort study	908 women ages 15–49 not using a method in 1992, interviewed two to three years later, and married to the same partner	Intention to use contraception in the future and timing of future intention to use	Intention to use in 1992 was a strong predictor of use between 1992 and 1995. Despite the strong association of intentions and use, more than one‐fourth of women surveyed behaved in a manner that was inconsistent with their intentions	Intention to use together with fertility preferences as person‐centered measurements can predict contraceptive behavior of reproductive‐aged women
Sarnak 2023	PMA countries	Examines intersections between intention to use and unmet need and how this relates to subsequent contraceptive behaviors	Cohort study, followed one year later	Noncontracepting women ages 15–49 at baseline, including pregnant and postpartum women	Interaction between unmet need and intention to use FP	Intention to use had more predictive power on use than unmet need	Proposes focusing on the intention to use and timing of that intention
Sarnak 2020	Uganda	Assesses the predictive utility of the unmet need measure on time to contraceptive adoption	Cohort study	747 noncontracepting women at baseline interviewed four years later	Concordance of unmet need and intention to use	Those with an intention to use are more likely to adopt over follow‐up, whether or not they have an unmet need	Intention to use a better measure than unmet need at the individual level
**Studies focused on adaptations to unmet need measure**							
Kuang 2014	DHS countries	Develop and define a direct measure of motivational intensity for contraception use	Cross sectional	Married/in union women not using any method in 23 countries in Africa	Creates eight groups of nonusers based on intentions, past use, and unmet need. Creates high motivation and low motivation groups	Motivation to use a contraceptive method links to certain socioeconomic traits. Low motivation nonusers are more rural, less educated, and closer to poverty. Important to consider nonusers in a more refined manner	Proposes using a combination of intention to use, past use, and unmet need status as proxy indicators of motivational intensity, a concept difficult to measure directly
Moreau 2019	DHS countries	Develops a point prevalence measure of unmet need for contraception and a point prevalence measure of unmet demand, incorporating intentions to use	Cross‐sectional	Women of reproductive ages	Current status unmet need and current status unmet demand that brings in the intention to use	Standard unmet need is higher than the current status unmet need. About one half of the current status unmet need group has no intention to use, that is, no demand for contraception	Need to consider intention to use as part of identifying those who have gaps in use
Karra 2022	DHS countries	Develops and tests a revised unmet need measure using existing data sources	Cross sectional	Women of reproductive age from 56 DHS surveys	Distinguishing those with ideal contraceptive prevalence from true contraceptive prevalence	Unmet need using the new counterfactual‐based measure is, on average, 5–6 percentage points higher than the standard measures of unmet need	Simplified measure, independent from biases generated through the use of reported preferences (as done with unmet need) and other problematic assumptions
Senderowicz 2022	DHS countries	Analyzes the contributions of supply‐side and demand‐side unmet needs	Cross sectional	Married and unmarried women of reproductive age with an unmet need	Supply‐side unmet need; demand‐side unmet need based on women's self‐reported reasons for nonuse	Demand‐side unmet need (lack of demand for contraception) is higher than the supply‐side unmet need (lack of access)	An indicator that overlooks a person's stated reproductive choice allows providers to influence decisions and can create incentives for coercion
Moore 2015	DHS countries	Develops a prospective unmet need approach with composite pregnancy risk and examines recent patterns among postpartum women	Cross sectional	Women ages 15–49 who had a birth in the last 0–23 months	Prospective definition of unmet need based on women's fertility preferences	Prospective unmet need among postpartum women is high (61 percent).	Slight adaptation to unmet need for postpartum women (prospective), but still based on unmet need
Sinai 2017	Mali and Benin	Develops an alternative unmet need measure that addresses individual's perceptions of his or her need	Cross sectional	1,080 (Benin) and 425 (Mali) women of reproductive age	Five current‐need status groups, each with different programmatic needs: real met need, perceived met need, real no need, perceived no need, and perceived unmet need	In Mali, the perceived unmet need was much higher. In Benin, perceived unmet need was low because women believed (incorrectly) that they were protected from pregnancy. Perceived no need was quite high in both countries	Proposes a simplified approach to measure current‐need status; based on fewer questions than full unmet need, but still ignores preferences
**Studies focused on adaptations to met need or demand satisfied measure among users**							
Rothschild 2021	Kenya	Examines if an alternative unmet need measure that incorporates method satisfaction leads to meaningfully different estimates of unmet need	Cohort study: short message service (SMS) text follow‐up weekly over 24 weeks	990 clients from public clinics, ages 18+ or 14+ with prior pregnancy, using modern reversible contraception, and had at least one follow‐up	Method satisfaction	If we include method dissatisfaction than those dissatisfied have an unmet need, unmet need goes up significantly	Proposes revising many components of unmet need and incorporating satisfaction Relates to commentary by Rominski and Stephenson (2019)
Rothschild 2023	Kenya	Determine trajectories in women's experiences of contraceptive method dissatisfaction over a six‐month follow‐up period	Cohort study: SMS text follow‐up weekly over 24 weeks	947 clients ages 18+ (or 14+ if prior pregnancy) from FP or maternal and child health (MCH) clinics using modern, reversible contraception followed up over 24 weeks	Created four groups: consistent satisfaction, increasing dissatisfaction, decreasing dissatisfaction, and consistently dissatisfied	Adverse contraceptive experiences are common even among those who continue using	Assuming contraceptive continuation is indicative of satisfaction is flawed. Need to understand and meet needs of dissatisfied users
Bullington 2023	Burkina Faso	Examine the prevalence of nonpreferred method use and barriers to preferred method use	Cross sectional	1,210 women of reproductive age using a method from two demographic surveillance sites	Nonpreferred method use	Discusses reasons for nonpreferred method use among 40 percent of users. Many would prefer to use a pill, calendar, or SDM, and not what they are currently using	No novel indicator but measures preference of method and barriers to use of the desired method Relates to commentary by Burke and Potter (2023)
Gausman 2025	Argentina, Ghana, and India	Explore the construct validity of the standard demand‐satisfied indicator to alternative definitions that incorporate women's own perceived demand, choice, and satisfaction	Cross sectional	Women ages 15–49 from household surveys (Ghana and India); women exiting facilities for reproductive and maternal health services in Argentina	Direct questions: (1) demand, if wanted to use an FP method to prevent pregnancy; (2) choice, if had a high degree of autonomy in decision‐making at last FP visit; and (3) satisfaction, if satisfied with the FP method currently using	Demonstrates that in two of the three countries, the standard measure of “demand satisfied” substantially overstates progress achieved in meeting contraceptive needs compared to the value of the alternative indicator proposed	Importance to consider demand, choice, and satisfaction as part of person‐centered measurement and when considering FP needs Relates to commentary by Rominski and Stephenson (2019)
**Studies focused on capturing pregnancy desires**							
Rocca 2022	United States	Uses Desire to Avoid Pregnancy (DAP) scale to examine prospective pregnancy preferences and contraceptive use	Cross sectional	2,601 women ages 18–44, nonpregnant and fertile	Desire to Avoid Pregnancy scale with three domains: desires, emotions, and practical consequences	A higher DAP score is associated with contraceptive use. No significant difference between methods used by DAP score	DAP may be a novel way to capture pregnancy preferences (considered more person centered)
Samari 2020	United States	Uses Desire to Avoid Pregnancy scale to examine pregnancy preferences and contraceptive use	Cross sectional	509 women ages 15–45, sexually active in the last year, seeking health services in a healthcare facility	Desire to Avoid Pregnancy Scale	Greater DAP scores are associated with more consistent contraceptive method use. No difference in DAP scores across different method types	DAP scale is designed to measure how one feels about pregnancy in the next three months and childbearing within a year; it does not address preferences beyond that timeframe
Geist 2019	United States	Explore Pregnancy Attitudes, Timing, and How important is pregnancy prevention (PATH) questions as they relate to contraceptive method use	Cross sectional	3,130 women ages 18–45 presenting to the clinic for new contraceptive services	Pregnancy Attitudes, Timing, and How important is pregnancy prevention (PATH) survey. Feelings about a hypothetical pregnancy (intentions, timing, and prevention)	The chances of choosing long‐acting reversible contraception (LARC) were reduced for those who would like to get pregnant in the next —two to five years compared to not desiring a future pregnancy, being uncertain, or intending to become pregnant in 5–10 years. No effect of the importance of pregnancy prevention on method choice	Expanding research questions and counseling to consider the multiple domains of PATH has the potential to enhance our understanding of clients' personal pregnancy preferences
Wolgemuth 2018	United States	Examines the relationship between pregnancy intention and attitude toward a hypothetical pregnancy and associations with current contraceptive use	Cross‐sectional: phone interview	858 Veterans ages 18–44 at risk of unintended pregnancy	Plans to get pregnant in the future and attitude toward hypothetical pregnancy	Both plans to get pregnant and attitudes toward pregnancy are associated with contraceptive use and method effectiveness	Measure pregnancy intentions and orientations toward a future pregnancy
Miller 2018	United States	Examine how positive and negative pregnancy desires affect the risk of pregnancy, including perceptions of partner desires	Cross sectional	854 women ages 18–19	How much do you want to get pregnant next month? How much do you want to avoid pregnancy in next month? Also, by the perception of partner's desires	Results support the conclusion that sexual and contraceptive behaviors mediate the effects of women's pregnancy desires and their perception of their partner's desires regarding the risk of pregnancy	No specific recommendations for measurement
**Studies proposing other novel measures**							
Senderowicz 2023	Burkina Faso	Test new survey items on contraceptive autonomy through the subdomains of informed choice, full choice, and free choice	Cross sectional	3929 women of reproductive age from two demographic surveillance sites	Measures contraceptive autonomy: informed choice, full choice, and free choice	Low levels of contraceptive autonomy are based on responding affirmatively to all three domains	Proposes strategies to measure informed, full, and free choice Relates to commentary by Senderowicz (2020)
Rothschild 2024	Nigeria	Examines Preference‐aligned fertility management (PFM) as an alternative to contraceptive use; also examines satisfaction‐adjusted PFM to describe concordance between current and desired contraceptive use	Cohort study: Phone or in‐person follow‐up at 3.5 months after initiation	1,020 young women age 15–19, currently married and newly initiating a modern method	Preference‐aligned fertility management (PFM) and satisfaction‐adjusted PFM	Among women who adopted a method in the last three months, 5 percent reported not wanting to use contraception; and half were currently using a contraceptive method despite not wanting to. 3 percent of the cohort were dissatisfied with their current method	Need to consider preferences and satisfaction as part of improved measures of FP Relates to commentary by Holt et al. (2023)
He 2017	United States	Describe contraceptive method preferences and use and preference‐use mismatch.	Cross sectional	414 women ages 18–55 at risk of pregnancy (users and nonusers)	Contraceptive method preference; mismatch between women's contraceptive method preference and use	Preference‐use mismatch was highest among less effective users. Cost/insurance concerns were among the top reasons for contraceptive preference‐use mismatch, closely followed by other access and provider‐related issues	Uses two questions to capture preferences and mismatch (and reasons for not using the preferred method) Relates to commentary by Burke and Potter (2023)
Gomez 2024	United States	Compare the conventional metric (use of effective contraception) to a person‐centered metric (use of preferred contraceptive method)	Cross sectional	Women ages 15–44, nationally representative sample	Conventional metric (use of effective contraception among survey respondents presumed to be at risk of unintended pregnancy); person‐centered metric (use of preferred contraceptive method—desire to maintain current contraceptive use among current and prospective users). Removes from the person‐centered measure those who are content nonusers—do not have a self‐identified need for contraception	More than half of users with both metrics are using their preferred method (51 percent conventional; 59 percent person‐centered). 13 percent of those in the conventional metric denominator do not want to use it. Conventional metric also misses many who are using but not at risk of pregnancy. Conventional metric also misses those who are not using the preferred method or who want to stop use	Measure method preferences for a broader set of individuals, including the preference not to use a method Relates to commentary by Burke and Potter (2023)

NOTE: Some studies say they include women of reproductive ages without specifying exact ages; typically, this is ages 15–49. DHS, Demographic and Health Survey; PMA, Performance Monitoring for Action.

^a^ Study sample focuses on the analytical sample rather than the full population surveyed in some settings.

These quantitative studies include a variety of study populations including women of reproductive age (e.g., in the ages 15–49); pregnant and/or postpartum women, including first time mothers (Borges, Santos, and Fujimori 2018; Gage, Wood, and Akilimali 2021; Moore et al. 2015); married and in some cases unmarried women; FP clients (Rothschild, Brown, and Drake 2021; Rothschild et al. 2023; Geist et al. 2019); and adolescents and/or youth (Khan, Tavrow, and Adamu 2023; Rothschild et al. 2024). The studies included come from a variety of countries, including a number that use cross‐country Demographic and Health Survey (DHS) data from multiple countries (n = 5) (Kuang et al. 2014; Moreau et al. 2019; Karra 2022; Moore et al. 2015; Senderowicz and Maloney 2022) or Performance, Monitoring and Action (PMA) data from multiple countries (Sarnak, Anglewicz, and Ahmed 2023). Eight studies come from the United States. Other countries included in the review are Argentina (*n* = 1), Bangladesh (*n* = 1), Benin (*n* = 1), Brazil (*n* = 1), Burkina Faso (*n* = 2), Democratic Republic of Congo (*n* = 1), Ghana (*n* = 2), India (*n* = 2), Kenya (*n* = 2), Mali (*n* = 1), Morocco (*n* = 1), Nigeria (*n* = 2), and Uganda (*n* = 2). Note that the number of countries is higher than the number of studies since two studies included multiple countries (Sinai, Igras, and Lundgren 2017; Gausman et al. 2025). Most studies found in the review were cross‐sectional (*n* = 20); the remaining studies followed a cohort over time, ranging from a 3.5‐month follow‐up to as much as six years between survey rounds. Below, we summarize the lessons learned from these studies to inform person‐centered FP measurement. Details of the 30 studies can be found in Table 3, which includes the location(s) of the study, the study design, the research objective, the study population, the person‐centered measures or concepts, the main results, and the relevance to measurement.

#### Intention to Use Studies

A recent scoping review from Boydell and Galavotti (2022) is on the measure of intention to use contraception with the view that this is a “more person‐centered measure of demand” because it more closely captures women's stated preferences around contraceptive use. The review finds many studies (*n* = 112) that focus on intention to use and points to an increase in the use of this measure since 2015. The authors identify three main ways that intention to use has been employed: (1) to augment or supplement the measure of unmet need; (2) to identify who may have gaps or needs for contraception to help inform behavior change communication programs; and (3) to predict future contraception adoption. The authors demonstrate widespread use of the measure of intention to use contraception, but they also demonstrate that there is no standard definition of the measure. The authors call for more work to clarify this “more person‐centered measure of demand” to better understand the complexity of the measures that underly it in relation to fertility intentions, contraceptive use intentions, and the intersection of these and how these varying intentions relate to a person's implementation of his or her short‐ and long‐range fertility and FP goals (Boydell and Galavotti 2022).

Our scoping review included many cross‐sectional and longitudinal papers included in the Boydell and Galavotti (2022) review. The studies found included some that were solely focused on intention to use at one point in time (Hanson et al. 2015; Gage, Wood, and Akilimali 2021; Khan, Tavrow, and Adamu 2023; Atiglo and Codjoe, 2019) while others examined how intention to use corresponded to later use of a method using longitudinal data (Callahan and Becker 2014; Lutalo et al. 2018; Borges, Santos, and Fujimori 2018; Roy et al. 2003; Curtis and Westoff 1996). Finally, two studies examined the intersection of intention to use and unmet need and how this corresponds to later use with longitudinal data (Sarnak et al. 2020, Sarnak, Anglewicz, and Ahmed 2023). The cross‐sectional studies focused on intention to use all bring in unique approaches to examine contraceptive use intentions including assessing whether perceived behavioral control (e.g., how easy or hard it is to use birth control every time one has sex, how likely one is to use birth control each time they have sex under various scenarios) is related to contraceptive use intentions (Hanson et al. 2015), determining the role of perceived norms and personal agency on postpartum FP use intentions of young moms (Gage, Wood, and Akilimali 2021), examining an index of empowerment and its association with contraceptive use intentions (Khan, Tavrow, and Adamu 2023), and examining the role of household decision‐making autonomy on contraceptive use intentions (Atiglo and Codjoe 2019). Each of the studies make recommendations for improved measurement around decision‐making, autonomy, or empowerment; however, they do not make unique recommendations for person‐centered FP measurement since they are simply examining whether women intend to use but are not linking this with their actual behaviors or perspectives on what the women themselves feel are preferable outcomes.

The longitudinal studies identified examine whether and how intentions to use contraception (and in some cases, intentions interacted with unmet need) predict subsequent contraceptive use. The studies find that while intention to use is predictive of subsequent contraceptive use in the short (six months) or longer (six years) follow‐up period, there remain a number of women who do not actualize their earlier intentions by the time of follow‐up (Lutalo et al. 2018; Borges, Santos, and Fujimori 2018; Curtis and Westoff 1996). These longitudinal studies are not inherently person‐centered given that they assume that the a person's contraceptive use intentions at an earlier point in time should be reflected in subsequent use without examining (a) if the person had changes in their contraceptive and fertility intentions, (b) if the person intends to use but chooses not to use, and (c) if the person adopted a method since the first observation but may not be using at the time of the follow‐up survey. The underlying assumption of the included studies is that there is a unidirectional positive outcome, subsequent use, which by nature, makes these studies not person‐centered. Finally, two studies (Sarnak et al. 2020, Sarnak, Anglewicz, and Ahmed 2023) examine unmet need and contraceptive use intentions and how each of these correlates with subsequent use. The authors demonstrate a stronger correlation between intentions and later use than unmet need and later use. These studies are not identifying person‐centered measures or approaches as they are still predicated on the assumption that intentions at an earlier point in time are meaningful for later behaviors and focus on increased contraceptive use as the “ideal” outcome, rather than considering one's own preferred outcome, which may change over time.

#### Adaptation of Unmet Need Studies

The set of studies that adapt unmet need all do so in varying creative ways that provide greater depth on who is “at risk” of a pregnancy. It is notable that while these studies seek to address some of the flaws identified with unmet need, none of these studies propose a person‐centered measure since they fail to capture individuals’ self‐defined needs, wants, and preferences. All but one of these studies use DHS data and, therefore, are limited in their ability to propose improved person‐centered measures. That said, some of the studies help to elucidate additional challenges with the unmet need measure and the importance of considering person‐centered framing. For example, Moreau and colleagues (2019) use DHS data and propose a point prevalence measure of unmet need for contraception as well as a point prevalence measure of unmet demand that incorporates intentions to use. The authors demonstrate that only about half of women with an unmet need intend to use contraception in the future; this is an important distinction for understanding future directions for FP programming with a more person‐centered framing (Moreau et al. 2019). Similarly, Senderowicz and Maloney (2022, 2024), using the standard unmet need definition, examine women's reasons for nonuse and create two categories: demand‐side unmet need and supply‐side unmet need. The authors identify that while this is not a person‐centered measure as it is based on the standard unmet need approach, it is more useful for considering barriers to use as it indicates that a sizeable share of women with an unmet need have a demand‐side unmet need, reflecting their lack of desire or need to use a method.

#### Adaptations to Met Need and Demand Satisfied Studies

Four studies were found that propose making adaptations to “met need” or “demand satisfied” with a particular focus on current contraceptive users. These studies are consistent with recommendations by Rominski and Stephenson (2019) and Burke and Potter (2023) in their commentaries that propose the importance of considering contraceptive method satisfaction and method preferences. Rominski and Stephenson (2019) propose that improved measures of contraceptive need should take into consideration a person's level of satisfaction with a method. They identify that many women may be using a method and thus have a “met need” but may be dissatisfied with their method and therefore have an “unmet need” for a different method. Likewise, Burke and Potter (2023) promote the importance of asking contraceptive users about their method preferences instead of just assuming that all women who are using a method are using their preferred method(s); this is considered a person‐centered approach. Burke and Potter (2023) demonstrate that across 12 studies that ask whether people are using their preferred method, between 18 percent and 67 percent of study samples report unsatisfied preferences (i.e., not using their preferred method); this may lead to contraceptive discontinuation. Thus, considering contraceptive preferences and satisfaction with methods are important considerations to ensure that measures truly identify what women, men, or adolescents need or want.

The four studies that adapt met need or demand satisfied begin to capture the components of person‐centered measurement; however, some continue to make assumptions about positive outcomes, for example, contraceptive use. Two of the studies by Rothschild, Brown, and Drake (2021 and 2023) were carried out in Kenya, where the authors recruited a sample of clients from public clinics who were using a modern method; these women were followed weekly to examine longitudinally satisfaction with their method. The authors use information about dissatisfaction to create updated estimates of unmet need that include those women who report being dissatisfied with their current method (Rothschild, Brown, and Drake. 2021). While this approach is more person‐centered by taking into consideration people's satisfaction with their method rather than assuming that all users are satisfied, it does continue to suffer from the same flaws as the unmet need measure of assuming that there is a “need” among those who are dissatisfied and not accounting for unwanted use of FP methods.

Bullington and colleagues (2023) examine the prevalence of nonpreferred method use and barriers to preferred method use in Burkina Faso. In this case, the authors demonstrate that many women are using a method that is not their preferred method; this is indicative of potential gaps in person‐centered FP programming. The authors do not propose a specific person‐centered measure to better assess gaps in programming; however, by asking women about their preferred method, they are already going beyond the assumption that all users are satisfied and have their needs met.

Finally, in a recent paper that covers Argentina, Ghana, and India, Gausman and colleagues (2025) examine a revised “demand satisfied” indicator that incorporates components of demand for contraception, decision‐making autonomy at last FP visit (choice), and satisfaction with the current method. The authors demonstrate that in two out of the three countries (Argentina and India), demand satisfied is overstated using the standard measure compared to their revised measure that captures components of demand, choice, and satisfaction. The revised measure of demand satisfied by Gausman and colleagues (2025) is simple because it uses the standard questions and only incorporates a small number of additional questions to measure demand and satisfaction. That said, it still misses the bar of being person‐centered since it makes assumptions that all users have a desire to use without explicitly asking directly who wants to use a method of contraception.

#### Studies on Pregnancy Desires

Five studies from the United States were found that examine pregnancy desires and contraceptive use behaviors and are trying to assess these with a more person‐centered care framing. Two of the studies (Rocca et al. 2022; Samari et al. 2020) use the Desire to Avoid Pregnancy (DAP) scale to examine prospective pregnancy preferences and contraceptive use. The DAP is a 14‐item scale that has three domains: desires, emotions, and practical consequences (Rocca et al. 2022). Both studies demonstrate that those with higher DAP scores were more likely to use contraception (Rocca et al. 2022) or use contraception more consistently (Samari et al. 2020). In both studies, there was no association between the DAP score and method choice, including the effectiveness of the method selected. The authors propose that the DAP is a novel way to capture how one feels about a future pregnancy in the next three months or childbearing in the next year, allowing for uncertainty and ambiguity in preferences; this is considered a more person‐centered approach than simply asking about future fertility desires, as is typically done as part of standard measures, including as part of the unmet need algorithm. The other three studies in this category employ differing approaches to measure pregnancy desires including employing the Pregnancy Attitudes, Timing and How important is pregnancy prevention (PATH) questions (Geist et al. 2019); examining attitudes toward a hypothetical pregnancy among veterans at risk of a pregnancy (Woldemuth et al. 2018); and examining positive and negative pregnancy desires among young women ages 18–19 (Miller, Barber, and Gatny 2018). None of these studies make specific recommendations for improved person‐centered measurement of FP demand and use.

#### Studies Proposing Novel Measures

Four studies were identified that propose novel person‐centered measures of fertility and FP needs, desires, and intentions. The first study employs an approach based on a commentary by Senderowicz (2020) that finds that reproductive agency is often ignored, and this needs to be considered for improved measurement and assessment of what people need or want in terms of contraceptive use. In this commentary, the author proposes a novel indicator called “contraceptive autonomy” that is measured through three subdomains—informed choice, full choice, and free choice (Senderowicz 2020). In an application of this approach, Senderowicz and colleagues (2023) test a set of survey items on contraceptive autonomy to capture the three subdomains using data collected from two surveillance sites in Burkina Faso. The authors identify that across the three subdomains, there are low levels of contraceptive autonomy if users and nonusers are meant to respond affirmatively across all three subdomains. That said, most users and nonusers report high free choice across the items assessed and lower informed and full choice; these are the factors that bring down the overall contraceptive autonomy scores. This paper provides specific items to capture contraceptive autonomy and its subdomains and is a step forward toward person‐centered FP measurement to understand women's experiences getting (or not) getting the contraceptive method(s) they prefer or want. That said, the items that make up the three subdomains still reflect the bias of what researchers or program managers believe are important for decision‐making, including knowledge of how to use methods, knowledge of advantages and disadvantages of methods, availability of a full range of methods, and lack of coercion in decision‐making. Not to say these are not important programmatic objectives but to truly assess person‐centered contraceptive autonomy, it is necessary to also ask women more broadly what they want and if they are able to attain their personal needs, wants, and preferences without predefining correct experiences or outcomes. Thus, while the contraceptive autonomy measure helps advance measurement of autonomy overall, it does not fill the role of specifically advancing person‐centered measurement.

The second novel measurement paper employs an approach proposed by Holt and colleagues in their 2023 commentary. In this commentary, the authors identify a novel person‐centered measure that they call Preference‐Aligned Fertility Management (PFM), which incorporates contraceptive use preferences. PFM requires asking women a small number of questions about whether they want to be using a method to avoid pregnancy; whether they are currently using a method to avoid pregnancy; and if they are using, if they want to be using their current method right now. With these questions, the proposal is to consider a woman to have PFM if she is currently using and reports that she is using the method she wants to use as well as among women who are currently not using and report that they desire not to use. The authors propose that this approach is more reflective of what women (or men or couples) want rather than judging what they need based on their “risk of pregnancy.” Further, the authors propose that they are neutral to method choice, whereby if a woman is choosing to use a traditional method, she too would have PFM. The authors are testing this approach in upcoming work in Nigeria and Uganda.

The PFM approach is incorporated into a study in Nigeria by Rothschild and colleagues (2024). In their study, the authors examine PFM as an alternative measure to contraceptive use and extend the PFM approach by measuring satisfaction‐adjusted PFM by asking if women are satisfied with their current method (Rothschild et al. 2024). The authors demonstrate that among women who adopted a method just over three months earlier, 5 percent report not wanting to use contraception, and half of these women were currently using a method despite not wanting to; these women do not have a preference aligned fertility management. Further, the authors demonstrate that 3 percent of women are dissatisfied with their method; these women also have underserved needs, whether to discontinue or switch methods. The authors promote the importance of considering preferences and satisfaction as part of improved FP measurement.

Third, a study from the United States among women of reproductive age (users and nonusers of a method) describes contraceptive method preferences and use to examine preference‐use mismatches (He et al. 2017). The authors demonstrate that there is more preference‐use mismatch among the less effective method users; this was often related to cost or insurance concerns that made it difficult to get one's preferred method. Additional explanations for the mismatch were related to provider factors that made it difficult to get one's preferred method. The authors propose the use of two questions to assess preferences and reasons for not using one's preferred method. Notably, this study was included in the commentary by Burke and Potter (2023) and has questions that they propose as relevant for improved person‐centered measurement.

Finally, a recent study by Gomez and colleagues (2024) uses data from a nationally representative sample of women to compare the conventional metric of “effective contraceptive use” used for national programming in the United States to a novel person‐centered metric. The conventional metric focuses on the use of effective methods^3^ as the numerator and puts all women who had ever had sex and are not currently pregnant or seeking pregnancy (i.e., at risk of pregnancy) in the denominator. The novel person‐centered metric is “use of preferred contraceptive method,” which is measured as the current contraceptive users who do not want to switch or stop using a method, as the numerator and the denominator are made up of all persons who are current or prospective users of contraception. This approach overlaps with the proposal by Burke and Potter (2023) to consider a person's method and use preferences. Gomez and colleagues (2024) demonstrate that across both the conventional and the person‐centered metrics, more than half of respondents are using their preferred method (51 percent conventional and 59 percent person‐centered). The authors demonstrate problems with the conventional metric, including that it misrepresents those who are not using their preferred method or who want to stop, and it includes in the denominator some people who do not want to use a method. At the same time, the novel metric is not fully person‐centered because it assumes the need for some populations (such as current contraceptive users) without explicitly asking what they want and need. Notably, the novel metric is still a considerable improvement over the conventional metric in terms of advancing person‐centered measurement.

## DISCUSSION

This scoping review provides an overview and analysis of how the concept of person‐centeredness has been employed to advance a new measurement of contraceptive need and use. For this review, we defined measures as person‐centered if they assess directly a person's preferences, wants, and desires while at the same time assessing if the person is able to or wants to meet those preferences, wants, and desires. At the same time, we also recognize that measures may exist on a continuum toward more person‐centeredness, and identify the progress being made with the novel metrics proposed.

Using search terms specific to FP and person‐centered framing, we identified many eligible articles to review; that said, only a small number made specific recommendations related to the measurement of FP needs, wants, and desires with a person‐centered lens. The studies found included commentaries pushing for novel measurement approaches and encouraging the field to move away from problematic measures that prioritize externally defined “pregnancy risk” or demographic targets over internally defined decision‐making autonomy related to fertility and FP (Speizer, Bremner, and Farid 2022; Fabic 2022; Dehlendorf et al. 2018).

The studies identified demonstrate the need for the FP measurement field to reconsider (a) what we are measuring, (b) for whom we are measuring. and (c) why we are measuring specific indicators. For example, a person‐centered framing of FP measurement considers method choice to be just that, a choice (Spagnoletti et al., 2019; Osei et al., 2014; Berglas et al., 2021). Therefore, if a person chooses to use a method and that method is a traditional method, this needs to be considered a “met need” or “satisfied user” or someone with “preference‐aligned fertility management” (Holt et al. 2023). Moreover, if a woman is using implants but is not satisfied with her method, she should be considered to have an “unmet need” or should not be considered to have a “met need” (Rominski and Stephenson 2019).

A clear understanding of for whom we are developing person‐centered FP measures is also crucial for identifying which measures make the most sense. Are we capturing outcomes for global monitoring, or are we seeking program‐specific outcomes that are amenable to short‐term changes? This also has implications for the number of questions that make sense to include to assess these person‐centered outcomes. For example, the contraceptive agency (Senderowicz et al. 2023), and the Contraceptive Intent Questionnaire (Raine‐Bennett et al. 2015) all have many items to capture relevant scales; these are useful for monitoring and evaluation of programs related to improving person‐centered care, decision‐making autonomy, method choice, and contraceptive continuation. That said, at the global monitoring level, more streamlined measures or questions need to be identified since these are typically examined across many country settings. Proposals that include a small number of additional questions to capture demand, choice, and satisfaction (Gausman et al. 2025; Rothschild, Brown and Drake 2021; He et al. 2017; Holt et al. 2023) among users (and nonusers) make the most sense for inclusion in large demographic surveys and for global monitoring.

Further, considering why we are using person‐centered FP indicators is also crucial for determining which indicators make the most sense. Different indicators point to different programmatic, policy, and funding needs. For example, a focus on intention to use contraception (or unmet need and intention to use) is attractive as it may indicate who may use a method of contraception if it is made easily accessible, available, at low cost, with a full choice of methods, including traditional methods. As discussed above, while intention to use (and intention to use among those identified as having “unmet need”) has been found to be associated with subsequent contraceptive use for some women (Callahan and Becker 2014; Curtis and Westoff 1996; Sarnak et al. 2020, Sarnak, Anglewicz, and Ahmed 2023), this is not a person‐centered measure since the underlying assumption is that the expected outcome is use, ignoring the many reasons that a person may choose not to use during a follow‐up period. The intention to use indicator (separately or in combination with unmet need) is being used to identify “gaps in service use”; however, this approach makes assumptions about individuals’ preferences without explicitly examining and understanding individuals’ circumstances, including the relational influencers that may affect decision‐making to use or not to use that are related to reproductive agency (Fabic et al. 2023).

A focus on satisfaction and preferences, two concepts that are similar but distinct, supports person‐centered programming and measurement. Identifying a person's preferences to use (or not) and for a specific method, including traditional methods, ensures that programs are addressing individually identified needs and preferences rather than externally defined risks (Burke and Potter 2023; Bullington et al. 2023; He et al. 2017). Further, understanding satisfaction with decisions (including decision to use or not to use and satisfaction with choice of method) means that programs (and measurement) consider that users may not be satisfied or may change their needs over time; all important factors to consider as part of person‐centered programming and measurement (Rominski and Stephenson 2019; Rothschild, Brown, and Drake 2021; Gausman et al. 2025). As the concepts of preference and satisfaction are built into new metrics, additional considerations should be addressed related to assessing preference strength and formation, and how agency may shape expectations of satisfaction.

While a number of proposed approaches were identified, including those that adapt existing measures and those that propose novel measures, there still remain a number of gaps in our understanding of what to prioritize to support person‐centered FP measurement, as can be seen in Table 1. First, some of the novel proposed approaches, including preference‐aligned fertility management (Holt et al. 2023; Rothschild et al. 2024) and contraceptive autonomy (Senderowicz 2020; Senderowicz et al. 2023), need further testing and refinement for determining their utility across different settings and their alignment with programmatic needs and global priorities. In addition, the contraceptive autonomy measure may need to be reworked to be more reflective of what individuals value over what researchers (or program people) value. Further, the proposed adaptation of demand satisfied with additional measures of demand, choice, and satisfaction (Gausman et al. 2025) requires further consideration related to underlying assumptions around demand as well as an examination into the choice component that is based on the Family Planning Autonomy in Decision‐Making (FP‐ADM) scale that includes seven questions (Gausman et al. 2023); this makes it more complicated for recommending as a global indicator. The two novel approaches from the United States (He et al. 2017; Gomez et al. 2024) focus on adapting measures and bringing in preferences to capture contraceptive needs; these approaches should be considered, tested, and validated for scenarios outside the United States.

Across the metrics assessed, we also find variation in the time frames of relevance; some focus on current use and unmet preferences (Holt et al. 2023; Rothschild et al. 2024), while others focus on hypotheticals (e.g., ask about preferences in the absence of any cost constraints; He et al. 2017), or future plans, as with intention to use in the next year (Sarnak, Anglewicz, and Ahmed 2023), intention to use until wanting another child (Lutalo et al. 2018), and intention to use at any time in the future (Sarnak et al. 2020; Curtis and Westoff 1996). What differentiates current preferences from hypothetical preferences is whether current constraints are accounted for in the question. For example, someone may want to use a method today and is not using because of their partner's opposition. That person could potentially say they do not want to use a contraceptive method today, but when asked if they would want to use a method today in the absence of constraints, they may say yes. In thinking through these new measures, the small nuances of time frames and whether questions are built within individuals’ current lived realities versus asked in the absence of constraints are important to explore further.

Before we make recommendations for moving forward and conclude, it is important to revisit the limitations of this review. As mentioned above, this review was limited at the time of the review by a lack of standardized language around person‐centered FP measurement of needs and use. This meant that many identified studies were not relevant following the title and abstract review. Given this lack of standardized language, it is likely that we missed several relevant articles on the topic. To address this, we incorporated some additional studies that were found through external searches. Second, given the broad definition of person‐centered measurement, the review included papers that seemed to fit but at the extraction phase fit less well; these are included here but do not significantly inform the recommendations. Lastly, person‐centered measurement is a rapidly evolving area, and new studies and recommendations are being identified and tested in a continuous manner. Thus, there are some new papers that are coming out that are likely to have been missed, and these will need to be considered going forward as we identify future directions for improved measurement.

This analysis demonstrated some big‐picture issues that need to be considered as we seek to make recommendations for person‐centered measurement of FP need and use. First, it is necessary to recognize that there may not be a clear “north star” in person‐centered indicators. This is because a person‐centered framing equates the choice to use with the choice to not use (or the choice to use a modern method with the choice to use a traditional method); this means that it is difficult to create indicators that are meaningful for setting programmatic or global targets or goals. Supporting person‐centered measurement in the FP field means supporting individuals to make choices that align with their current (and evolving) needs, desires, and preferences. Given that these preferences rightfully change over the life course, it is important to identify measures that are meaningful and reflective of individuals’ evolving preferences. Second, while in this review we used a definition of person‐centered FP measurement that is currently getting some traction (Rothschild et al. 2025), we recognize that this is a newly proposed definition that still requires validation and adoption by the field. It is therefore unsurprising that this review indicated that we lack standard definitions and interpretations in the identified measures. This includes how we define intention to use contraception (Boydell and Galavotti 2022); whether we are focused on agency and/or autonomy and how we define autonomous decision‐making (Fabic et al. 2023); and how or whether we consider fertility and pregnancy preferences and intentions as part of person‐centered FP measurement (Rocca et al. 2022). Third, as seen in Table 1, gaps remain in developing, validating, and testing person‐centered measures around decision‐making and autonomy, pregnancy and fertility preferences, method preferences, and method satisfaction. Much of what has been examined has focused on intention to use contraception (and unmet need) using DHS or PMA data; additional questions, indicators, and data sources, including routine data sources such as District Health Information Software 2 (DHIS2), should be considered for improving person‐centered measures of FP.

Do the challenges identified above mean we should not measure person‐centered FP needs and use? Of course not. At this moment, there is a lot of interest in improved FP measurement, and as shown here, there have been several papers that consider the conceptual importance of person‐centered measurement, but only a limited number of proposed novel indicators. With that in mind, there is a need to undertake qualitative studies to better understand what outcomes are meaningful to women, men, couples, and adolescents related to their fertility, FP, and contraceptive decision‐making. The objective is to identify measures that reflect and respect people's own values, preferences, and decisions about FP. We need to better understand what is valued—not by researchers and program implementers, but by people themselves, and what they want for their reproductive lives and futures. And to do this, we need measures that ask people directly what they want for their own bodies and lives. This is consistent with the World Health Organization's framework on integrated people‐centered health services^4^ that seeks to identify and meet the health and well‐being needs of individuals throughout their life. Our review identified some important novel indicators and approaches that begin to move us toward person‐centered FP measurement; these should continue to be pursued while additional work is undertaken to strengthen our understanding of what it means to measure reproductive autonomy, contraceptive autonomy, and person‐centered FP needs, wants, and desires.

## CONFLICTS OF INTEREST STATEMENT

The authors report no conflicts of interest.

## ETHICS APPROVAL STATEMENT

Not applicable for this scoping review.

## PATIENT CONSENT STATEMENT

Not applicable for this scoping review.

## Supporting information



Supporting Information

## Data Availability

Not applicable for this scoping review.

## Footnotes

1 Report of Expert Group Meeting on Assessing Approaches to Demand‐Side Family Planning Measurement with a Reproductive Justice and Rights Framework (https://iussp.org/sites/default/files/RethinkingFP_Mombasa‐Final%20report.pdf).

2 Note that one study counted as scale development in Table 1 (Senderowicz et al. 2023) is discussed in the results under survey or quantitative data as it uses quantitative data and proposes a novel measure related to one of the commentaries (Senderowicz 2020).

3 Vasectomy, implant, IUD, injectable, oral contraceptive pill, the patch, the ring, or the diaphragm.

4
https://www.who.int/health‐topics/integrated‐people‐centered‐care#tab=tab_3
