# Supplementary material for: The State of Person‐Centered Measurement for Family Planning Need and Use: A Scoping Review
Source: Stud Fam Plann. 2025 Jun 11;56(3):403–36. doi: 10.1111/sifp.70019 (PMC12501745; doi:10.1111/sifp.70019)
Supplement: Supplementary file 1 — Supporting Information [file SIFP-56-403-s001.docx]

TABLE A1 Classification of studies into the relevant domains of contraceptive use and measure development stages

| **Measure**  **Development**  **Stages** | **Domains of Contraceptive Use** | | | | |
| --- | --- | --- | --- | --- | --- |
|  | *Decision-Making/Agency* | *Contraceptive Use*  *Intentions/Unmet Need*  *Related* | *Pregnancy/Fertility Preference* | *Method*  *Preference* | *Method Experience/Satisfaction* |
| *Conceptual - general* | Speizer, Bremner, Farid (2022); Fabic (2022); Senderowicz (2000); Dehlendorf et al (2018) | | | | |
| *Conceptual – specific concept* |  | Karra (2022)  Senderowicz & Maloney (2022)  Sinai et al. (2017) |  | Burke and Potter (2023)  Holt et al (2023) | Rominski & Stephenson (2019) |
| *Formative* | Harrington et al (2021)  Willan et al (2020)  Downey et al (2017)  Paul et al (2018) Spagnoletti et al (2018) | Boydell and Galavotti (2022)  Hayer et al (2022) | Burgess et al (2022)  Gomez et al (2021)  Grillo et al (2018) | Berglas et al (2021)  Osei et al (2014)  Spagnotletti et al (2019) |  |
| *Scale development* | Carvajal (2020)  Senderowicz (2023) | Raine-Bennett & Rocca (2015) |  | Senderowicz (2023) |  |
| *Validation* |  |  | Rocca et al (2022)  Samari et al (2020)  Geist et al (2019) |  | Gausman et al (2025) |
| *Applied – cross-sectional*  *Applied - longitudinal* | Atiglo & Codjoe (2019) | Hanson et al (2015)  Gage et al (2021)  Khan et al (2023)  Atiglo & Codjoe (2019)  Kuang et al (2014)  Moreau et al (2019)  Borges et al (2018)  Curtis & Westoff (1996)  Lutalo et al (2018)  Sarnak et al (2020)  Sarnak et al (2023)  Calahan et al (2014)  Roy et al (2003) | Moore et al (2015)  Wolgemuth et al (2018)  Miller et al (2018) | He et al (2017)  Bullington et al (2023)  Gomez et al (2024)  Rothschild et al (2024) | Rothschild et al (2023)  Rothschild et al 2021) |
